# Efficacy and long-term outcomes of montgomery T-tube placement in benign subglottic stenosis

**DOI:** 10.3389/fmed.2026.1741311

**Published:** 2026-02-09

**Authors:** Yishan Lv, Faming Liu, Bing Xue, Jie Zhang, Ting Wang

**Affiliations:** 1 Department of Respiratory and Critical Care Medicine, Beijing Tian Tan Hospital, Capital Medical University, Beijing, China; 2 Department of Respiratory and Critical Care Medicine, Chuiyangliu Hospital Affiliated to Tsinghua University, Beijing, China

**Keywords:** airway management, benign subglottic stenosis, decannulation, interventional bronchoscopy, long-term outcomes, montgomery T-tube, retrospective study

## Abstract

**Objective:**

To evaluate the efficacy and long-term outcomes of Montgomery T-tube placement in patients with benign subglottic stenosis.

**Methods:**

We retrospectively analyzed the clinical data of 45 patients with benign subglottic stenosis treated with Montgomery T-tube placement at Beijing Tian Tan Hospital and Beijing Chuiyangliu Hospital between May 2015 and May 2025. The etiologies of tracheal stenosis were as follows: Iatrogenic in 41 patients (91.11%), including 29 patients (64.44%) with post-tracheotomy tracheal stenosis (PTTS) and 12 patients (26.67%) with post-intubation tracheal stenosis (PITS); post-traumatic in 1 patient (2.22%); post-tuberculosis in 2 patients (4.45%); and associated with chronic obstructive pulmonary disease (COPD) in 1 patient (2.22%).

**Results:**

All 45 patients successfully underwent T-tube placement. Among them, 39 patients achieved sustained airway patency and restored phonatory function following T-tube insertion, defined as successful retention (39/45, 86.67%). Six patients required tube removal within 2 weeks due to granulation tissue proliferation or mucus plug obstruction, defined as retention failure (6/45, 13.33%). Intraoperative complications primarily included mucosal lacerations (12 cases, 26.67%), all of which healed spontaneously within 1 week postoperatively. Short-term postoperative complications mainly comprised subglottic edema (31 cases, 68.89%), mucus plug obstruction (24 cases, 53.33%), and granulation tissue proliferation (10 cases, 22.22%). At the end of follow-up, 14 of the 39 patients (35.9%) were successfully decannulated and achieved long-term airway stability. The cumulative median tube retention time was 20.5 months. Of the 39 patients, 25 (64.10%) continued to require long-term T-tube retention. Multivariate regression analysis further indicated that a higher Cotton–Myer grade was independently associated with a significantly lower likelihood of successful decannulation (*OR* = 0.168, 95%CI: 0.031–0.920, *P* < 0.05).

**Conclusion:**

Montgomery T-tube placement is a safe and effective interventional treatment for benign subglottic tracheal stenosis. It provides durable airway patency and functional improvement, particularly when surgical reconstruction is not feasible.

## 1 Introduction

Subglottic stenosis is defined as a narrowing of the tracheal segment between the inferior margin of the vocal cords and the inferior margin of the cricoid cartilage. It may cause varying degrees of dyspnea and even life-threatening asphyxia in severe cases. It remains a challenging clinical entity to manage and is often associated with a relatively poor prognosis (1). Benign subglottic stenosis is generally classified into congenital and acquired types, with the latter being more common. In China, the main causes of acquired subglottic stenosis include trauma, iatrogenic injury, and tracheobronchial tuberculosis, while other causes, such as benign tumors, infection, tracheal foreign bodies, and idiopathic stenosis, are less frequent. Surgical resection remains the definitive curative approach but is often limited by high cardiopulmonary demands, significant operative trauma, and the risk of postoperative complications (2, 3).

Bronchoscopic interventional therapies such as balloon dilation, cryotherapy, and laser therapy are widely used in the management of subglottic stenosis. When repeated interventions fail to achieve satisfactory and sustained outcomes, airway stent placement is often considered. Nonetheless, long-term stent implantation may result in complications such as migration and granulation tissue formation, which can significantly affect therapeutic efficacy. Compared with other airway stents, including metallic (uncovered or covered) and silicone stents, the Montgomery T-tube confers specific advantages for the long-term management of benign subglottic stenosis. These advantages include a lower propensity for stent migration, less granulation tissue formation, and better patient tolerance, which together facilitate airway hygiene and preservation of phonatory function (4, 5). Nevertheless, most previous studies have primarily focused on short-term outcomes, while evidence regarding long-term efficacy and decannulation rates remains limited. This retrospective study aimed to evaluate the long-term outcomes and prognostic factors of Montgomery T-tube placement in patients with benign subglottic stenosis treated at Beijing Tian Tan Hospital and Chuiyangliu Hospital.

## 2 Subjects and methods

### 2.1 Study subjects

We retrospectively reviewed the clinical records of patients with benign subglottic stenosis who were admitted to the Department of Respiratory and Critical Care Medicine at Beijing Tian Tan Hospital and Chuiyangliu Hospital between May 2015 and May 2025. The inclusion criteria included:

① patients diagnosed with benign subglottic stenosis based on medical history, imaging studies, bronchoscopy, and pathological examination.
② Patients who underwent Montgomery T-tube placement at our hospital for subglottic stenosis.
③ Patients with complete clinical data. A total of 45 patients were enrolled, comprising 29 males and 16 females, with an age range of 14–74 years.

### 2.2 Methods

Patient medical records, imaging studies, and interventional bronchoscopy reports were reviewed to collect baseline characteristics, imaging findings, and bronchoscopic features. Statistical analyses were performed to evaluate the etiology, clinical characteristics, outcomes of T-tube placement, complications, and long-term prognosis in patients with benign subglottic stenosis.

#### 2.2.1 T-tube placement

All patients had undergone tracheostomy before the procedure. Under general anesthesia with muscle relaxation, assisted ventilation was provided via a disposable tracheostomy cannula connected to the anesthesia machine. A rigid bronchoscope was introduced orally. The stenotic segment above the tracheostomy stoma was dilated using techniques such as electrocautery and balloon dilation (for patients with airway atresia, it was necessary to first penetrate the atretic segment with a puncture needle). Subsequently, the rigid bronchoscope was advanced to the level of the tracheostomy stoma and connected to high-frequency jet ventilation. The original tracheostomy tube was removed, and the T-tube was inserted through the cutaneous tracheostoma, ensuring the upper limb of the T-tube was at least 5 mm below the vocal cords. After placement was completed, the rigid scope was removed, and ventilation was switched to a laryngeal mask airway connected to the anesthesia machine.

#### 2.2.2 T-tube removal

T-tube removal was performed as an inpatient procedure in the operating room. Before decannulation, preoperative assessment with chest computed tomography (CT) and bronchoscopy was conducted to verify airway structural stability, confirm the absence of significant residual stenosis, and ensure no substantial granulation tissue remained. All removals were conducted under general anesthesia, with complete airway emergency equipment immediately available. A lubricant was applied around the tracheostomy stoma, and the external limb was gently withdrawn until both its proximal and distal ends were withdrawn from the trachea. After removal, flexible bronchoscopy was performed to assess airway stability and patency to ensure patient safety. Following decannulation, the tube was replaced with a corresponding model of a metallic tracheostomy tube. The patient was observed for 24 h with the tube occluded. If no dyspnea occurred, the metallic tracheostomy tube was removed, and the fistula was closed by occluding the fistula (6, 7).

#### 2.2.3 Interventional therapies before T-tube placement

In this study, 27 patients received prior interventional treatments, including balloon dilation of the stenotic segment, electrocautery for scar tissue release, and cryotherapy for granulation tissue removal, aiming to alleviate stenosis and restore airway patency. Among them, one patient with diffuse tracheal stenosis secondary to endobronchial tuberculosis underwent multiple balloon dilations without satisfactory improvement. Two temporary airway stents (bare-metal and covered-metal) were subsequently placed but failed to maintain patency and were later removed. Another patient developed tracheomalacia with airway collapse after intubation and had an hourglass silicone stent inserted, which was later removed due to stent migration. One additional patient had previously undergone T-tube placement but required removal because of postoperative subcutaneous emphysema.

#### 2.2.4 Preoperative evaluation

To date, no unified international criteria exist for defining complex subglottic airway stenosis (CSAS). Accordingly, drawing on prior literature and clinical practice, we defined CSAS in this study as subglottic stenosis characterized by a stenotic segment ≥10 mm in length, complicated by unstable features including tracheomalacia, dynamic collapse, or airway atresia (1, 8).

#### 2.2.5 Airway stenosis characteristics and treatment outcome measures

##### 2.2.5.1 Stenosis type

Airway stenosis was classified into two categories:

① Anatomical Stenosis: including intraluminal growth, cicatricial contraction, tortuous airway, and extrinsic compression.
② Dynamic Stenosis: including excessive dynamic airway collapse and tracheomalacia.

##### 2.2.5.2 Stenosis degree

The Cotton-Myer grading system was used to assess the severity of tracheal stenosis, which is based on the percentage of airway obstruction (9): Grade I: obstruction ≤ 50%. Grade II: obstruction 50%−70%. Grade III: obstruction 71%−99%. Grade IV: obstruction 100% (lumen obliteration).

##### 2.2.5.3 Stenosis length

The length of the stenotic segment was calculated as: Stenosis length = (number of CT slices from the proximal to the distal margin of the stenosis) × (slice thickness).

##### 2.2.5.4 Tracheal wall thickness

Tracheal wall thickness is measured directly on CT images.

##### 2.2.5.5 Dyspnea and voice function

Dyspnea severity and vocal function were assessed using the modified Medical Research Council (mMRC) dyspnea scale and the Voice Handicap Index (VHI-10), respectively (10, 11).

### 2.3 Follow-up

Follow-up was conducted from May 2015 to May 2025. After T-tube placement, patients received clinical evaluations at 1, 3, and 6 months. Surveillance bronchoscopy was performed every 3 months. In-hospital follow-up centered on direct airway visualization in the operating room, which was essential for initial stent assessment, complication management, and decannulation evaluation. Patients without significant symptoms who adhered to proper tube care could continue follow-up at local healthcare facilities. In these cases, information was collected via structured questionnaires and review of external bronchoscopy reports and imaging studies. A dedicated research coordinator verified data consistency and supplemented any missing information to ensure the integrity of the dataset.

### 2.4 Statistical analysis

Statistical analysis was performed using SPSS version 27.0. Continuous variables with a normal distribution were expressed as mean ± standard deviation (SD), and comparisons between groups were conducted using the Student's *t*-test. Continuous variables that did not follow a normal distribution were expressed as median (interquartile range, IQR), and intergroup comparisons were performed using the Wilcoxon signed-rank test. Categorical variables were presented as frequencies and percentages (%), and group comparisons were conducted using Fisher's exact test.

Multivariate logistic regression analysis was performed to identify factors independently associated with successful T-tube decannulation. Candidate variables were selected a priori based on clinical relevance, established pathophysiological considerations, and prior literature, rather than solely on statistical significance in univariate analyses. Given the limited sample size and number of outcome events, the number of variables included in the final multivariate model was restricted to reduce the risk of overfitting and model instability. Variables demonstrating substantial collinearity or unstable estimates were excluded from the final model. All statistical tests were two-tailed, and a *P*-value <0.05 was considered statistically significant.

## 3 Results

### 3.1 General information

A total of 45 patients who met the inclusion criteria were included in the analysis. The demographic data, baseline characteristics, and airway stenosis features are summarized in Tables 1, 2. The cohort comprised 29 males and 16 females, with an age range of 14 to 74 years and a mean age of 47.76 ± 17.13 years. The interval from tracheostomy to the onset of stenosis ranged from 0.5 to 52 months, with a mean duration of 5.76 months. The interval from endotracheal intubation to the onset of stenosis ranged from 0.3 to 3 months, with a mean duration of 1.03 months. Following a comprehensive assessment that included preoperative three-dimensional reconstruction of chest computed tomography (CT), accurate bronchoscopic measurements, and intraoperative direct observation, none of the patients in this study showed obvious structural damage or collapse of the cricoid cartilage. Among the 45 patients, 16 cases (35.56%) fulfilled the diagnostic criteria for complex stenosis. At the early stage of disease, airway stenosis was categorized into anatomical, dynamic, or mixed types based on the pattern of obstruction. With disease progression, most patients eventually developed tracheomalacia with luminal collapse, which was the primary indication for Montgomery T-tube placement.

**Table 1:** Clinical baseline characteristics of the patients.

**Variables**	***N* = 45**
**Gender (%)**	
Male	29 (64.44%)
Female	16 (35.56%)
Age (years, mean ± SD)	47.76 ± 17.13
**Comorbidities**	
Cardiovascular disease	21
Diabetes	9
Cerebrovascular disease	5
Other	8
**Cause of intubation or tracheostomy (%)**	
Infection	5 (11.11%)
Cardiovascular disease	4 (8.89%)
Cerebrovascular disease	14 (31.11%)
Traumatic brain injury	10 (22.22%)
Neck trauma	2 (4.44%)
Thyroid enlargement	3 (6.67%)
Acute exacerbation of chronic lung disease	4 (8.89%)
Other	4 (8.89%)

**Table 2:** Characteristics of airway stenosis.

**Variables**	***N* = 45**
**Types of stenosis (%)**	
**Anatomical stenosis (%)**	
Intraluminal growth	1 (2.22%)
Cicatricial contraction	23 (51.11%)
Tortuous airway	4 (8.89%)
Extrinsic compression	0
**Dynamic stenosis (%)**	
Excessive dynamic airway collapse	1 (2.22%)
Tracheomalacia	9 (20.00%)
Mixed type (%)	7 (15.56%)
**Severity of the stenosis (%)**	
Cotton-Myer II	9 (20.00%)
Cotton-Myer III	17 (37.78%)
Cotton-Myer IV	19 (42.22%)
**Length of stenosis (%)**	
<1 cm	7 (15.56%)
1–3 cm	30 (66.67%)
3–5 cm	5 (11.11%)
>5 cm	3

### 3.2 Success and failure of T-tube placement

All 45 patients successfully underwent T-tube placement, achieving a procedural success rate of 100%. Among them, 43 patients had successful placement on the first attempt, resulting in an initial placement success rate of 95.56% (43/45). Of the two patients who failed the initial placement, one patient underwent preoperative electrocautery and balloon dilation to release scar tissue. The first attempt failed due to a narrow tracheostomy stoma and airway collapse above the stoma. Bronchoscopy revealed a tracheal mucosal laceration, which led to the abortion of the procedure. After 1 week of anti-infective therapy, a second attempt was made but resulted in partial obstruction when the upper limb of the T-tube embedded into the laceration. A temporary tracheostomy tube was placed, and after 2 weeks of healing, the T-tube was successfully inserted on the third attempt. The other patient had severe subglottic granulation tissue overgrowth, causing airway obstruction, which required repeated endotracheal intubation and mechanical ventilation during hospitalization. Significant dynamic airway collapse was observed during this period. During the initial T-tube placement, the patient's oxygen saturation progressively decreased, necessitating temporary insertion of a tracheostomy tube. After subsequent bronchoscopic interventions, a second attempt at T-tube placement was performed successfully.

### 3.3 Efficacy evaluation

Wilcoxon signed-rank tests indicated that both the mMRC dyspnea scores and VHI-10 voice handicap scores were significantly reduced after stent placement compared with preoperative values (*P* < 0.01). Detailed results are presented in Table 3.

**Table 3:** Comparison of pre- and postoperative mMRC grades and VHI-10 scores (*N* = 39).

**Variable**	**Preoperative**	**Postoperative**	***Z*-value**	***P*-value**
mMRC grade	3 (2, 4)	1 (0, 2)	−5.56	<0.01
VHI-10 score	30 (14, 40)	9 (6, 14)	−5.10	<0.01

Data are presented as median (interquartile range, IQR = P25, P75). Given that the data deviated from a normal distribution based on normality tests, the Wilcoxon signed-rank test was used for comparisons. P <0.05 was considered statistically significant.

### 3.4 Complications

Intraoperative complications (*n* = 45): All patients experienced mild bleeding ( ≤ 20 ml). Tracheal mucosal lacerations occurred in 12 patients (26.67%), all of which healed spontaneously within 2 weeks postoperatively.

Short-term postoperative complications (*n* = 45): These included subglottic edema (31 cases, 68.89%), mucus plugging of the lumen (24 cases, 53.33%), granulation tissue formation (10 cases, 22.22%), postoperative infection (10 cases, 22.22%), transient fever (14 cases, 31.11%), and hoarseness (6 cases, 13.33%).

Long-term postoperative complications (*n* = 30): Among patients requiring long-term T-tube placement, complications included T-tube migration (2 cases, 5.13%), granulation tissue formation (18 cases, 50%), and mucus plugging (9 cases, 25%).

All complications were managed using standard clinical interventions, including bronchoscopy-guided removal of granulation tissue, adjustment or replacement of T-tubes, antimicrobial therapy, and other symptomatic treatments as appropriate.

### 3.5 Follow-up and re-evaluation

In this study, among patients who achieved successful T-tube placement and long-term retention, a total of 62 voluntary hospital visits were recorded during follow-up. Of these, 44 were scheduled follow-ups for regular evaluation or decannulation assessment, while 18 were unplanned visits due to infection, restenosis, or other complications. Most visits occurred more than 12 months after placement, highlighting the importance of sustained long-term surveillance in this population.

Among the scheduled visits, 28 were performed for routine clinical evaluation. Ten visits (22.73%) resulted in successful T-tube removal after in-hospital evaluation. In nine visits (20.45%), airway examination showed that decannulation was not yet feasible, and the T-tube was replaced. Another nine visits (20.45%) involved airway restenosis after T-tube removal, requiring reinsertion to restore airway patency. 16 visits (16/44, 34.09%) were conducted for airway secretion management, during which patients underwent suctioning, removal of mucus plugs with cotton swabs or brushes, or bronchoscopic debridement of granulation tissue.

Among the unscheduled revisits, 13 (72.22%) were related to bacterial colonization or newly developed airway infections. These patients received antibiotic therapy combined with bronchoscopic interventions for the clearance of purulent secretions and necrotic debris. Two unscheduled revisits occurred due to hoarseness and glottic edema, appearing at 3 and 12 months after T-tube placement, respectively. After removal, trimming of the superior limb, and debridement of granulation tissue at both ends of the tube, the T-tube was reinserted, and the symptoms subsequently resolved.

### 3.6 Decannulation time

Among the 45 patients, 6 (13.33%) underwent decannulation 4–27 days postoperatively due to inadequate ventilation with the T-tube, and these patients subsequently received a metallic tracheostomy tube to maintain airway patency. After maintaining stable airways for 1 year with a T-tube, 1 patient was re-evaluated and confirmed to meet surgical criteria, and subsequently underwent tracheostomy reconstruction surgery. 14 patients (31.11%) were successfully decannulated after 12–79 months following confirmation of airway stability. The median total indwelling time for this group was 20.5 months. The remaining 25 patients (55.56%) continued long-term T-tube placement at the end of the study, necessitating regular tube changes. Their cumulative indwelling time ranged from 13 to 119 months, with a median of 70 months.

## 4 Discussion

With the increasing prevalence of pulmonary tuberculosis in China and the widespread use of endotracheal intubation and tracheostomy, the incidence of benign subglottic airway stenosis has risen (12, 13). In this study, iatrogenic injury was identified as the predominant cause of stenosis, accounting for 91.11% (41/45) of cases. Among these, post-tracheostomy stenosis accounted for 64.44% (29/45) and post-intubation stenosis for 26.67% (12/45). Abnormal healing at the tracheostomy site, excessive cuff pressure, and prolonged mechanical ventilation serve as primary contributors (14–16). Therefore, standardization of intubation techniques, avoidance of repeated or traumatic intubations, preferential use of high-volume, low-pressure cuffs, optimization of airway management, and minimizing the duration of mechanical ventilation are essential measures to reduce the risk of airway stenosis (17, 18).

Compared with malignant airway stenosis, the management of benign subglottic stenosis is often more challenging and prone to long-term complications. Although surgical resection of the stenotic tracheal segment followed by end-to-end anastomosis remains the definitive treatment, stringent patient selection remains essential. In the present cohort, patients who did not undergo the target surgical procedure were classified into five categories:

① Long-segment or complex stenosis, for which achieving a tension-free anastomosis was technically unfeasible (*n* = 3, 6.67%).
② Pre-existing tracheomalacia or dynamic airway collapse, in which anastomotic support would be inadequate, thereby increasing the risk of anastomotic leakage or fistula formation (*n* = 17, 37.78%).
③ Stenosis involving or adjacent to the subglottic region, associated with a substantial risk of postoperative vocal dysfunction as well as excessive anastomotic tension (*n* = 11, 24.44%).
④ Severe medical comorbidities (e.g. heart failure or advanced renal insufficiency), in which multidisciplinary team (MDT) evaluation by thoracic surgeons and anesthesiologists determined that the perioperative risk was unacceptably high (*n* = 5, 11.11%).
⑤ Patient refusal of surgery after comprehensive counseling regarding potential risks and anticipated benefits (*n* = 9, 20.00%). These findings illustrate the practical considerations underlying surgical decision-making in benign subglottic stenosis.

For patients who are not candidates for surgical intervention, bronchoscopic interventional therapy serves as an effective alternative for maintaining airway patency. Given its acceptable safety profile and low complication rate, the Montgomery T-tube remains a widely used option for the management of benign subglottic airway stenosis (19, 20).

In this study, all 45 patients with benign subglottic stenosis achieved successful Montgomery T-tube placement. Those with long-term T-tube retention showed marked improvement in respiratory and vocal functions compared to preoperative status. Initial placement failure was associated with anatomical factors and inadequate preoperative airway preparation, including narrow tracheostomy stoma, mucosal lacerations, and dynamic tracheal wall collapse (21). Therefore, thorough preoperative evaluation of airway anatomy and targeted preparatory interventions are essential for optimizing placement success and minimizing procedural complexity (22, 23).

Montgomery T-tube placement demonstrated reliable therapeutic efficacy with minimal invasiveness and few contraindications. Early complications are primarily related to the procedure itself, including mucosal laceration, glottic edema, and, in rare cases, subcutaneous emphysema or pneumothorax. Such complications typically resolve with postoperative prophylactic antimicrobial therapy and budesonide administration for edema reduction.

Long-term complications were typically associated with stent presence, such as granulation tissue formation, lumen obstruction due to mucus or necrotic debris, and tube migration (24, 25).

Our findings indicate that postoperative granulation tissue formation at both ends of the stent is primarily associated with mechanical irritation, airway infection, and individual susceptibility to keloid formation. Granulation most frequently developed at the superior limb of the T-tube. Therefore, comprehensive preoperative assessment of airway stenosis was essential for selecting an appropriate T-tube length and diameter. Previous research suggested that maintaining a T-tube-to-trachea diameter ratio of approximately 0.78 minimizes airway wall compression and reduced the risk of granulation formation (26, 27).

It was generally recommended that the T-tube length should extend approximately 3 mm to 1 cm beyond both the proximal and distal margins of the stenosis. After trimming, the superior and inferior edges should be smoothly beveled to minimize mucosal trauma. In addition, the upper limb should be positioned at least 1.5–2 cm below the glottis to minimize vocal cord stimulation, alleviate glottic edema, and prevent granulation development (19).

Other studies have also identified retained airway secretions as another significant factor contributing to T-tube placement failure. Therefore, comprehensive preoperative assessment and meticulous postoperative care—including regular nebulization therapy to facilitate sputum clearance and maintain airway humidity—were crucial in preventing mucus plugging and subsequent stent failure (28).

Patients with benign airway stenosis typically have a long life expectancy, making standardized long-term management after T-tube placement essential (29–31). For patients requiring long-term T-tube retention, adherence to standardized care protocols played a crucial role in reducing the risks of mucus plugging and infection. Therefore, consistent standardized management after discharge can effectively reduce the frequency of unplanned hospitalizations, improved long-term quality of life, and facilitated the ultimate goal of decannulation. Standardized long-term management consisted of 3 major components: lifestyle adjustments (smoking cessation and alcohol restriction, moderate exercise, adequate hydration for mucus clearance, and avoiding abrupt head-neck movements), T-tube maintenance (limiting unnecessary external limb opening to prevent foreign body inhalation, plus thrice-daily nebulization to avoid luminal mucus plugs), and regular follow-up (bronchoscopy every 1–3 months with scheduled T-tube replacement) (8, 32).

There is currently no established consensus on the optimal timing for T-tube removal. Previous studies have suggested that decannulation can be attempted 18–24 months after placement (33). In this study, 14 patients underwent regular bronchoscopic evaluation and tube replacement following successful T-tube placement. Comparative pre- and post-placement CT imaging of the neck and chest showed significant thinning of the tracheal wall at the stenotic segment during long-term follow-up (*t* = 2.983, *P* = 0.011; detailed results are presented in Table 5). In addition, partial separation of the T-tube from the surrounding airway wall was observed, indicating stabilization of the tracheal structure. Successful decannulation was achieved in these patients after 12–79 months, representing successful long-term airway stabilization, and the overall decannulation rate reached 35.90% (14/39). Nine patients developed significant tracheal softening and collapse upon attempted T-tube removal, requiring reinsertion. All nine had evidence of pre-existing airway softening or dynamic collapse before initial tube placement. Therefore, the observed restenosis was interpreted as reflecting persistent airway structural instability rather than a complication directly induced by T-tube removal.

Most existing studies recommended that maintaining T-tube placement for at least 1 year; however, extending the indwelling time beyond this does not appear to improve decannulation outcomes. Muraleedhara et al. (34) conducted an 18-month study and found that tracheal scar tissue tends to stabilize approximately 3 months after placement. Their findings showed no significant difference in decannulation success rates between short-term (<3 months) and long-term (>6 months) T-tube placement groups, suggesting a possible pathological basis for early decannulation.

Therefore, the timing of T-tube removal should be individualized. Before attempting decannulation, it is essential to conduct a comprehensive assessment of the relationship between the T-tube and the surrounding tracheal wall, as well as the overall stability of the airway, to ensure that the lumen remains patent after tube removal. For patients with keloid-prone scarring or tracheomalacia with dynamic airway collapse, prolonged indwelling for more than 2 years—or even permanent placement—was sometimes necessary (35).

At the end of follow-up, 25 patients still required long-term T-tube retention because CT imaging and tracheoscopic evaluation revealed granulation tissue formation around the T-tube with tight adhesion to the surrounding tracheal wall, indicating an unstable airway structure. We therefore adopted a more conservative extubation strategy, thus prolonging the T-tube indwelling duration. The cumulative indwelling time ranged from 13 to 119 months, with a median indwelling time of 70 months. The non-decannulation rate was 64.10% (25/39). Multivariable logistic regression analysis identified that a higher Cotton-Myer grade was independently associated with a significantly lower likelihood of successful decannulation (*OR* = 0.168, *P* = 0.040 <0.05). Detailed results are presented in Table 4. Previous studies have confirmed that multiple factors influence successful T-tube decannulation. Tsakiridis K et al. (36) reported that lesion type was a key predictor of successful decannulation. The absence of cardiovascular disease history and early T-tube placement within 6 months post-intubation were independent predictors of successful decannulation. Another study that included 49 patients with post-tracheostomy tracheal stenosis, using multivariable Cox proportional hazards regression analysis, identified diabetes mellitus and cerebrovascular disease as independent risk factors for prolonged T-tube indwelling. The underlying mechanism may involve a localized hyperglycemic wound environment in diabetic patients, which delays macrophage transition from a pro-inflammatory to an anti-inflammatory phenotype and inhibits the release of pro-healing factors (37). Furthermore, patients younger than 30 years and those without airway cartilage involvement were more likely to achieve successful decannulation. The type of primary disease, the length of stenosis, and the circumference of tracheal wall involvement were also associated with T-tube decannulation outcome (38, 39).

**Table 4:** Univariable and multivariable logistic regression analyses of factors associated with successful decannulation.

**Factor**	**Univariable analysis**		**Multivariable analysis**	
	**OR (95% CI)**	***P*** **-value**	**OR (95% CI)**	***P*** **-value**
Cotton-Myer Grade (per grade increase)	0.387 (0.146-1.023)	0.056	0.168 (0.031-0.920)	0.040
Age (per year increase)	1.008 (0.974-1.044)	0.636	0.998 (0.949-1.050)	0.949
**Gender**				
Male (reference)	1.00	–	1.00	–
Female	2.444 (0.542-11.028)	0.245	3.916 (0.568-26.973)	0.166
**Diabetes mellitus**				
No (reference)	1.00	–	1.00	–
Yes	0.864 (0.179-4.161)	0.855	2.751 (0.329-22.977)	0.350
**Cardiovascular disease**				
No (reference)	1.00	–	1.00	–
Yes	0.667 (0.178-2.491)	0.547	0.779 (0.140-4.333)	0.775
**Etiology of stenosis**				
Non-iatrogenic (reference)	1.00	–	1.00	–
Tracheostomy	0.707 (0.184-2.724)	0.614	0.695 (0.032-15.178)	0.817
Intubation	1.176 (0.281-4.926)	0.824	0.750 (0.035-15.895)	0.853
Duration of stenosis (per month)	0.963 (0.736-1.259)	0.781	0.698 (0.431-1.131)	0.145
**Regular nebulization and care**				
No (reference)	1.00	–	1.00	–
Yes	2.000 (0.345-11.583)	0.439	1.303 (0.146-11.624)	0.813

OR, odds ratio; CI, confidence interval. The outcome event is successful decannulation. *P* < 0.05 was considered statistically significant.

**Table 5:** Comparison of tracheal wall thickness before T-tube insertion and before decannulation (*N* = 14).

**Comparison**	**Mean difference ±SD (mm)**	**95% CI for difference**	***t*-value**	***df***	***P*-value**
Before T-tube insertion vs. before decannulation	0.63 ± 0.79	0.17 – 1.09	2.983	13	0.011

Data are presented as mean ± standard deviation. *P* < 0.05 was considered statistically significant. The paired-sample *t*-test was used for comparisons, after confirming the normality of the difference scores with the Shapiro-Wilk test. The data met the assumption of normality based on the Shapiro–Wilk test.

This study has several inherent limitations. It uses a retrospective design and features a relatively small sample size. These factors restricted statistical analysis and comprehensive evaluation of T-tube removal-related factors. Our 10-year follow-up data provide clear evidence that the Montgomery T-tube placement is safe for long-term airway management. It proves effective in patients with complex subglottic stenosis ineligible for radical surgical reconstruction. Extensive lesions, unstable airway anatomy, or severe comorbidities drive this ineligibility. For this specific patient population, the primary therapeutic goal is defined. It focuses on preserving airway patency and enhancing the quality of life. Isolated T-tube decannulation is not the intended endpoint. Future studies require larger cohorts and multicenter collaboration. Such a design will yield more robust clinical evidence. It will also further refine clinical decision-making and treatment strategies.

## 5 Conclusion

In summary, Montgomery T-tube placement offers a safe and effective long-term airway management solution for patients with benign subglottic stenosis, particularly when surgical reconstruction is not feasible. Combined with standardized follow-up and postoperative care, this procedure ensures sustained airway patency and achieves significant functional improvement.

## Data Availability

The original contributions presented in the study are included in the article/supplementary material, further inquiries can be directed to the corresponding author.
